# Comparative metabolomics reveals organ-specific discrepancy in TCMSP-predicted bioactive ingredients between two geographically distinct regions of *Rehmannia chingii*

**DOI:** 10.7717/peerj.20722

**Published:** 2026-02-03

**Authors:** Wanbo Zhang, Xinjie Jin, Ying Zhang, Luhan Peng, Haifeng Wang, Yongqun Chen, Yonghua Zhang

**Affiliations:** 1 College of Life and Environmental Science, Wenzhou University, Wenzhou, Zhejiang, China; 2 Guoxi Middle School, Ouhai District, Wenzhou, Zhejiang, China

**Keywords:** *Rehmannia chingii*, Metabolome, Comparative analysis, Organ-specific, Pharmaceutical function, Geographical origin

## Abstract

**Background:**

The geographical region and organ-specific accumulation of metabolites in medicinal plants are critical determinants of their pharmaceutical efficacy. *Rehmannia chingii*, an endemic species native to eastern China and a significant member of the genus *Rehmannia*, exhibits multiple bioactive properties in both its leaves and roots. However, spatial distribution of its pharmaceutical ingredients across various geographical regions remains inadequately understood.

**Methods and Results:**

This study combined widely targeted metabolomics with the Traditional Chinese Medicine System Drug Analysis Platform (TCMSP) to investigate the accumulation patterns of medicinal ingredients in the leaves and roots of fresh *R. chingii* from two distinct geographical regions. Among the 1,420 metabolites identified, four differential biomarkers were identified: *p*-coumaroylcadaverine and protocatechuic acid-4-*o*-glucoside, which were primarily associated with geographical differentiation, and 5, 6-dimethyl-2-benzofuran-1, 3-dione and daphnin, which were indicative of organ type classification. Additionally, 31 potential bioactive ingredients were prioritized *via* TCMSP screening. Metabolic profiling further revealed that multiple flavonoids were enriched in leaves, whereas roots accumulated higher levels of tangeretin, 6-*o*-*p*-coumaroylajugol, guanosine, virexilactone, and aucubin. Notably, coniferin and tangeretin, with oral bioavailability values ≥30% and drug-likeness values ≥0.18, were identified as key potential bioactive marker ingredients, and they were highly abundant in *R. chingii* from the Tianmu Mountain region of Hangzhou.

**Conclusion:**

These findings highlight the critical role of geographic and organ-specific factors in determining the metabolic profiles of *R. chingii*, thereby advancing our understanding of its medicinal value and providing a theoretical basis for the rational exploitation and utilization of its medicinal resources.

## Introduction

Recent studies have increasingly focused on the abundant metabolic components found in medicinal plants, which not only constitute fundamental constituents of drugs but are also directly associated with their therapeutic effects (Zhao et al., 2023b). A series of investigations have established connections between plant secondary metabolism and environmental factors in geo-herbalism, highlighting pharmacophylogenetic patterns and underscoring the necessity for species-specific metabolomic analyses (Alami et al., 2024). Variations in growing conditions and developmental organ types are recognized as significant determinants of metabolite accumulation (Li et al., 2020). For example, the paeoniflorin identified in *Semiliquidambar cathayensis* demonstrates considerable variation based on its geographic origins and the specific organs analyzed (Tian et al., 2023). Similarly, the roots of *Astragalus membranaceus* are particularly rich in polysaccharides and flavonoids, whereas the stems and leaves contain fewer medicinal compounds (Tian et al., 2025a). Likewise, *Lycium barbarum* fruits are abundant in polysaccharides and carotenoids, while the leaves exhibit relatively low levels of secondary metabolites (Alessandra et al., 2018). Consequently, geographic origins significantly influence the metabolite content in traditional Chinese medicinal plants, with distinct organs imparting unique characteristics to the medicinal properties of different plant parts.

*Rehmannia chingii,* a member of the genus *Rehmannia* within the family Orobanchaceae, is a well-known traditional Chinese medicinal plant primarily distributed in Zhejiang Province, China, with additional populations found in southern Anhui and northern Jiangxi, making it a typical species endemic to eastern China (Albach et al., 2006; Han et al., 2024). While *R. chingii* possesses documented medicinal value (Wang et al., 2025b; Liu et al., 2016), its congener *R. glutinosa* has been more extensively studied for therapeutic applications, including the treatment of chronic diseases such as cancer, cardiovascular diseases, diabetes, depression, and Alzheimer’s disease (Zhou et al., 2018; Zhou et al., 2016). To date, over 200 bioactive compounds have been detected, including iridoids, ionones, phenyl ethanols, triterpenoids, flavonoids, lignans, phenolic acids, and other compounds (Chen et al., 2021). In contrast, only a limited number of compounds, including one iridoid and phenethyl alcohol glycosides, have been isolated and identified in *R. chingii* (Liu et al., 2016). Moreover, the specific distribution of metabolites across different geographical regions and plant organs in *R. chingii* remains poorly understood. Variations in the concentration and types of pharmaceutical ingredients across different regions and organ types can significantly impact medicinal efficacy and safety (Jian et al., 2015; Wang et al., 2025a). Furthermore, the absence of a comprehensive comparative metabolomic analysis of *R. chingii* hinders the development of standardized protocols for the extraction and utilization of its bioactive compounds, which is critical for optimizing its medicinal applications.

In the realm of traditional Chinese medicine research, the integration of widely targeted metabolomics and network pharmacology presents a novel approach to elucidate the components of plant-based drugs (Zhao et al., 2024). Metabolomics provides comprehensive insights into the chemical constituents of plants and their metabolic pathways within organisms, thereby enhancing drug design and elucidation of mechanisms (Manickam et al., 2023). When combined with network pharmacology, these methodologies afford a more thorough understanding of the pharmacological effects of traditional Chinese medicines. For example, this integrated approach has been employed to analyze the metabolites of specific herbal formulas, revealing their potential therapeutic mechanisms across various diseases (Zhang et al., 2023). Overall, this comprehensive strategy not only identifies drug components but also clarifies their interactions and biological impacts through network analysis.

Therefore, this study aims to conduct a comprehensive metabolome-based comparative analysis of the leaves and roots of *R. chingii* from two distinct regions, in conjunction with the Traditional Chinese Medicine System Drug Analysis Platform (TCMSP) database. The primary objectives of this research are threefold: (1) to perform thorough metabolomic profiling of *R. chingii* and its differential synthesis pathways; (2) to identify and quantify the pharmaceutical ingredients that exhibit discrepancies specific to geographic regions and organ types; and (3) to preliminarily screen potential targeted metabolites in relation to documented medicinal functions. By achieving these objectives, this study aspires to enhance the scientific understanding of the complex metabolome of *R. chingii* and inform the development of more effective and safer medicinal applications.

## Materials & Methods

### Plant collection

**Figure 1:**
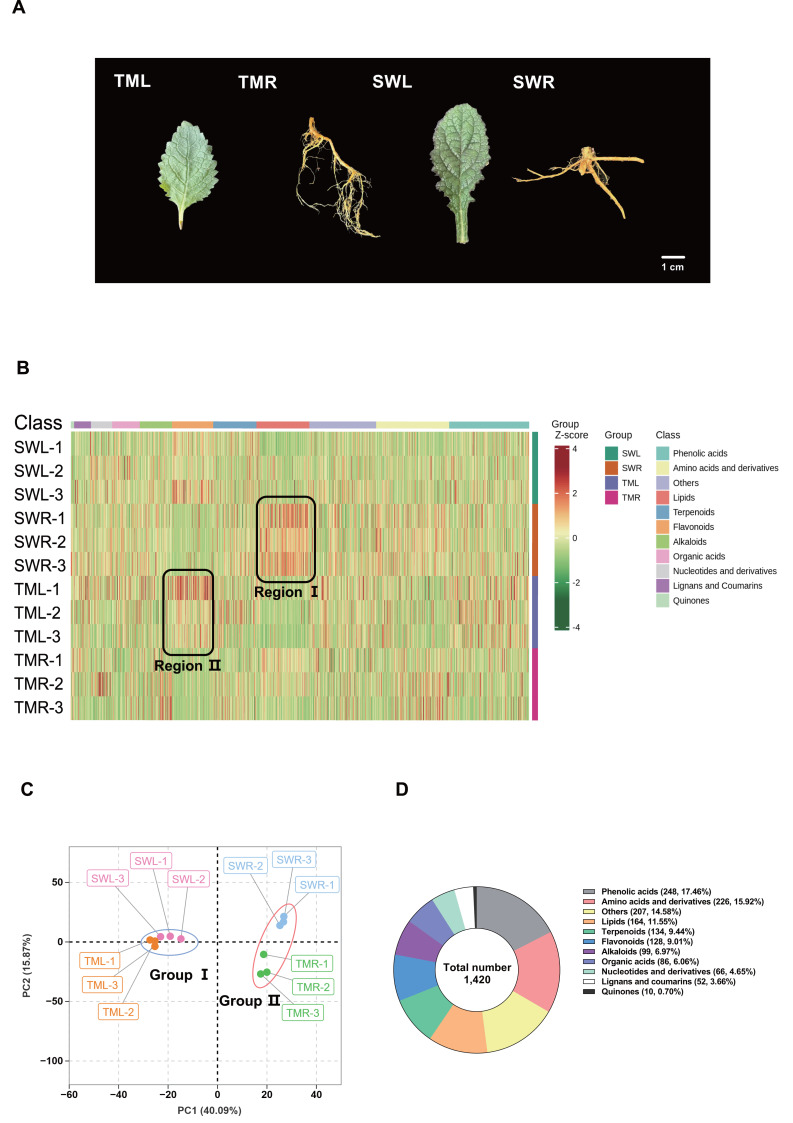
The sample information and widely targeted metabolic components analysis. (A) Photographs of fresh *R. chingii* leaves and roots collected in two regions (B) The hierarchical cluster analysis (HCA) of all detected metabolites. The *x*-axis represents the 11 categories of compounds and the *y*-axis represents the sample names. After standardization, the calculated values of different relative contents are shown from red to green, which represent the highest to lowest levels. The distinct difference regions were framed by black dashed lines. (C) Principal component analysis (PCA) based on the organ-specific metabolic components. (D) Statistics and percentage of all identified metabolites. SWL and SWR were leaves and roots collected in Songwan village, respectively. TML and TMR were leaves and roots collected in Tianmu mountains, respectively. The two distinct groups were marked by red and blue circles.

Over ten wild samples of *R. chingii* were collected during the natural flowering period in April 2023. The primary ecological characteristics at each site include Tianmu Mountain (30°19.31′N, 119°26.92′E, 300–400 m altitude, montane forest habitat) in Lin’an, Hangzhou, and Songwan Village (27°51.43′N, 120°25.82′E, 20–30 m altitude, agricultural margin habitat) in Rui’an, Wenzhou, Zhejiang Province, China. Subsequently, to minimize transient stress and preserve genetic-based geographic signals, these samples were transplanted to a common garden at Wenzhou University, where homogenized loam soil was employed for one month. Fresh leaves and roots were then selected for experiments and designated as Tianmu Mountain leaves (TML), Tianmu Mountain roots (TMR), Songwan Village leaves (SWL), and Songwan Village roots (SWR) (Fig. 1A). All test materials were authenticated as *R. chingii* by Prof. Yonghua Zhang (Wenzhou University) based on the key diagnostic trait of purple-red corollas (Wang et al., 2025b), which distinguishes the flowers of *R. glutinosa*.

### Sample preparation and extraction

Sample preparation and extraction methods were performed according to *Alami et al. (2023)*. Initially, 50 mg of lyophilized powder derived from fresh samples was combined with 1,200 μL of a pre-cooled internal standard, which consisted of a 70% methanol-water extract at −20 °C. Subsequently, the organic mixture was shaken six times, with each shaking lasting 30 s and a 30 min interval between each shaking. After centrifugation at 12,000 rpm for 3 min, the supernatant of each sample was carefully collected. The samples were then filtered using a microporous filter membrane with a pore size of 0.22 µm and stored in a sample bottle for UPLC-MS/MS analysis.

### Metabolite detection

The chromatographic detection methods were adapted from the procedure described by *Li et al. (2022)*. Sample extracts were detected *via* ultra-performance liquid chromatography (UPLC) (Exion LC™ AD, https://sciex.com.cn/) combined with tandem mass spectrometry (MS/MS). The UPLC analytical conditions included an Agilent SB-C_18_ column (1.8 µm, 2.1 × 100 mm); the mobile phase consisted of solvent A (pure water with 0.1% formic acid) and solvent B (acetonitrile with 0.1% formic acid). The separation utilized a gradient elution program, starting with a composition of 95% A and 5% B, transitioning over 9 min to 5% A and 95% B, which was maintained for 1.0 min. This was followed by reverting to 95% A and 5% B over the course of 1.1 min, and this final composition was sustained for 14 min. The flow rate, column oven temperature, and injection volume were set at 0.35 mL/min, 40 °C, and 2.0 μL, respectively. The operational parameters for electrospray ionization (ESI) included a source temperature of 500 °C; an ion spray voltage (IS) of 5,500 V for positive ion mode and −4,500 V for negative ion mode; and ion source gas I (GSI), gas II (GSII), and curtain gas (CUR) were set at 50, 60, and 25 psi, correspondingly. QQQ scans were executed in multiple reaction monitoring (MRM) mode, utilizing a medium collision gas (nitrogen). The declustering potential (DP) and collision energy (CE) for specific MRM transitions were refined through further optimization.

Quality control (QC) samples were created by combining sample extracts to evaluate the consistency of sample analysis under identical treatment conditions. Typically, during the instrument analysis phase, a single QC sample is added for every 10 samples that are detected and analyzed to ensure the reliability of the analysis process. The repeatability of metabolite extraction and detection, which reflects the feasibility of technical repetition, can be assessed by examining the overlap in the total ion current (TIC) profiles of mass spectrometry detection across various QC samples (Figs. S2, S3).

### Coefficient of variations from the *R. chingii* samples

The coefficient of variation (CV) represents the ratio of the standard deviation of the original dataset to its mean and serves as a measure of data variability. The empirical cumulative distribution function (ECDF) is useful for assessing the frequency of substances featuring CVs below a specified reference threshold. In a QC sample, a larger fraction of substances with lower CVs indicates increased reliability of the experimental results. In this investigation, over 85% of the substances exhibited CV values under 0.5, suggesting robust stability of the experimental data. Furthermore, more than 75% of the QC samples had CV values below 0.3, reinforcing the conclusion that the experimental results were extremely stable (Fig. S4).

### Qualitative and quantitative compounds information

Qualitative analysis was conducted through a proprietary Metware (MWDB) database to compare retention times (RT), secondary mass spectra (all fragment ions in the material), molecular weights of precursor ions, and characteristic molecular weights of fragments. We matched ions, DP, and CE to data in the materials database. We also excluded isotope signals; repetitive signals containing K^+^, Na^+^, NH_4_^+^ ions; and repetitive signals of fragment ions of other higher molecular weight substances. Metabolites in Table S1 marked with an asterisk (*) are isomers that could not be structurally distinguished by UPLC-MS/MS.

The identified metabolites were quantified using triple quadrupole mass spectrometry in multiple reaction monitoring (MRM) mode (Fig. S5). In MRM mode, the source (parent) ions of the target material are first filtered through the quadrupole to eliminate interference from other material ions. Precursor ions were then fragmented into product ions in a collision chamber, followed by filtering with QQQ to select characteristic targets (Fig. S6). This eliminated interference from non-target ions, making quantitation more accurate and reproducible. After obtaining the spectra of metabolites from various samples, the mass spectral peak areas of all substances were integrated to calibrate the mass spectra of the same metabolite across different samples. The relative content of compound obtained was calculated using unit variance (UV) scaling.

Analyzing the correlation among samples allows for the examination of biological replicates within a group. Additionally, a higher correlation coefficient among samples within the same group, compared to that between different groups, indicates greater reliability of the identified differential metabolites. To assess the repeatability of biological correlations, the Pearson correlation coefficient *r* (commonly known as Pearson’s Correlation Coefficient) serves as a key evaluation metric. This coefficient was derived using R software’s built-in cor function. A value of —*r*— that approaches 1 signified a stronger correlation between two repeated samples (Fig. S7).

### Prediction of pharmaceutical ingredients

The identified metabolites of two *R. chingii* species and the related disease information of the corresponding targets were referenced from the database of the TCMSP (https://tcmsp.91medicine.cn). The listed ingredients with oral bioavailability (OB) ≥ 1% and drug similarity (DL) ≥ 0.01 in *R. chingii* samples from two sites were considered as potential bioactive ingredients in traditional Chinese medicine (TCM) (Li et al., 2022; Cao et al., 2023). Thresholds of oral bioavailability (OB ≥ 30%) and drug-likeness (DL ≥ 0.18) were selected based on TCMSP guidelines for prioritizing clinically relevant compounds (Ru et al., 2014). Compounds with OB <30% were excluded as they typically exhibit poor *in vivo* absorption, while DL <0.18 indicates low structural similarity to approved drugs.

### Kyoto Encyclopedia of Genes and Genomes (KEGG) annotation and enrichment analysis

The identified metabolites were annotated with the KEGG database (https://www.kegg.jp/kegg/compound/) and additionally using the KEGG pathway database (https://www.genome.jp/kegg/pathway.html). The process of annotation for these metabolites was carried out. Following this, a metabolite enrichment analysis was performed to illustrate the pathways associated with these significantly regulated metabolites. The significance was established according to the *p*-value obtained from the hypergeometric test.

### Data statistics and analysis

The hierarchical cluster analysis (HCA) of all metabolites was conducted using R software with the Complex Heatmap package. A pie chart illustrating the classification of compounds was generated using GraphPad Prism 9. Additionally, principal component analysis (PCA), orthogonal partial least squares discriminant analysis (OPLS-DA), *K-* means analysis, bar charts of differential metabolites, and volcano plots were constructed using R software (version 3.5.0, http://www.r-project.org/). In the analysis comparing two groups of samples, differential metabolites were identified based on variable importance in projection (VIP) scores (with VIP ≥ 1) and the absolute values of Log_2_ fold change (—Log2FC— ≥ 1.0). The structural formula of the identified pharmacological ingredient was created using Chem Draw (version 20.0). All samples were detected for three biological replicates. For metabolomic analysis, 10 independent adult plants were sampled from each site. Each biological replicate (*n* = 3 per group) was composed of pooled tissues from 5 distinct plants to ensure representativeness. All samples were randomized during metabolite extraction and UPLC-MS/MS detection to eliminate batch effects.

## Results

### Comparative metabolomics analysis of *R. chingii*

To comprehensively identify the chemical profiles of *R. chingii* organs from two distinct collection regions (Fig. 1A), we analyzed the methanol extracts using a widely targeted metabolomics approach with UPLC-MS/MS. The clustering heatmap of all detected compounds showed two distinctly separate groups consisting of the primary metabolism-lipid components of SWR (region I) and the secondary metabolism-flavonoid components of TML (region II) (Fig. 1B). The PCA results further demonstrate that the leaves collected from the two sites were clustered into Group I, implying their similar chemical composition. In contrast, the samples from Group II were clearly separated, indicating the obvious discrepancy in root components (Fig. 1C). Additionally, the optimized metabolomic workflow enabled robust detection of 1,420 metabolites across four samples with QC analysis confirmed high reproducibility. They covered 12 major classes, such as phenolic acids, flavonoids, terpenoids, accounting for the majority of known secondary metabolites in the *Rehmannia* genus (Chen et al., 2021; Liu et al., 2016), confirming comprehensive coverage. Apart from 226 amino acid derivatives and 164 lipids as the primary metabolites, we also identified a wealth of secondary metabolites, including 248 phenolic acids, 134 terpenoids, 128 flavonoids, 99 alkaloids, 86 organic acids, 66 nucleotide derivatives, 52 lignans and coumarins, and 10 quinones in the roots and leaves of fresh *R. chingii* collected from two sites (Fig. 1D; Table S1).

**Figure 2:**
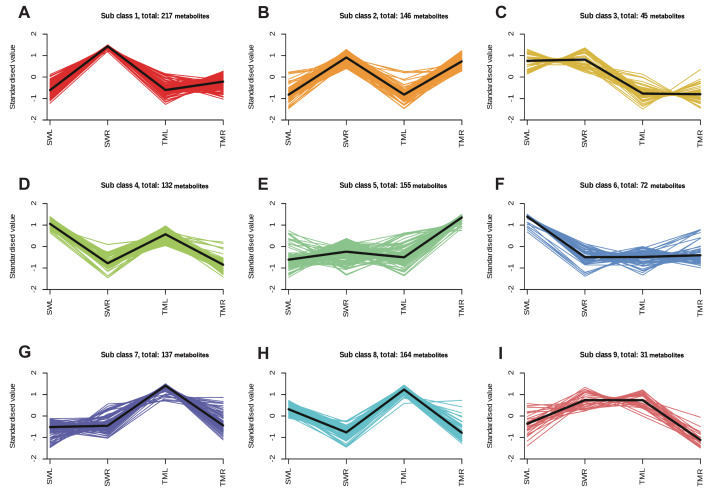
*K*-means clustering algorithm analysis map of all identified metabolites from two organs of the *R. chingii* species. SWL and SWR means the leaves and roots collected in Songwan Village, and TML and TMR means the leaves and roots collected in Tianmu Mountain. Lines of different colors represent the accumulation patterns of identified metabolites in different samples, with all metabolites enriched in a subset.

*K*-means clustering analyses were conducted to categorize the standardized values of all metabolites (Fig. 2). Among the nine subcategories, the results for organ-specific subcategories revealed that SWR and SWL contained 217 and 72 metabolites with high accumulation levels, respectively (Figs. 2A, 2F). In contrast, 155 and 137 metabolic compounds with higher concentrations were detected in TMR and TML, respectively (Figs. 2E, 2G). Additionally, the region-specific subcategories, illustrated in Figs. 2B and 2D, demonstrate that 146 and 132 compounds with higher concentrations were collected from the roots and leaves, respectively, at two distinct sites.

### OPLS-DA analysis of geographically and organ-specific differentially significant metabolites in *R. chingii*

**Figure 3:**
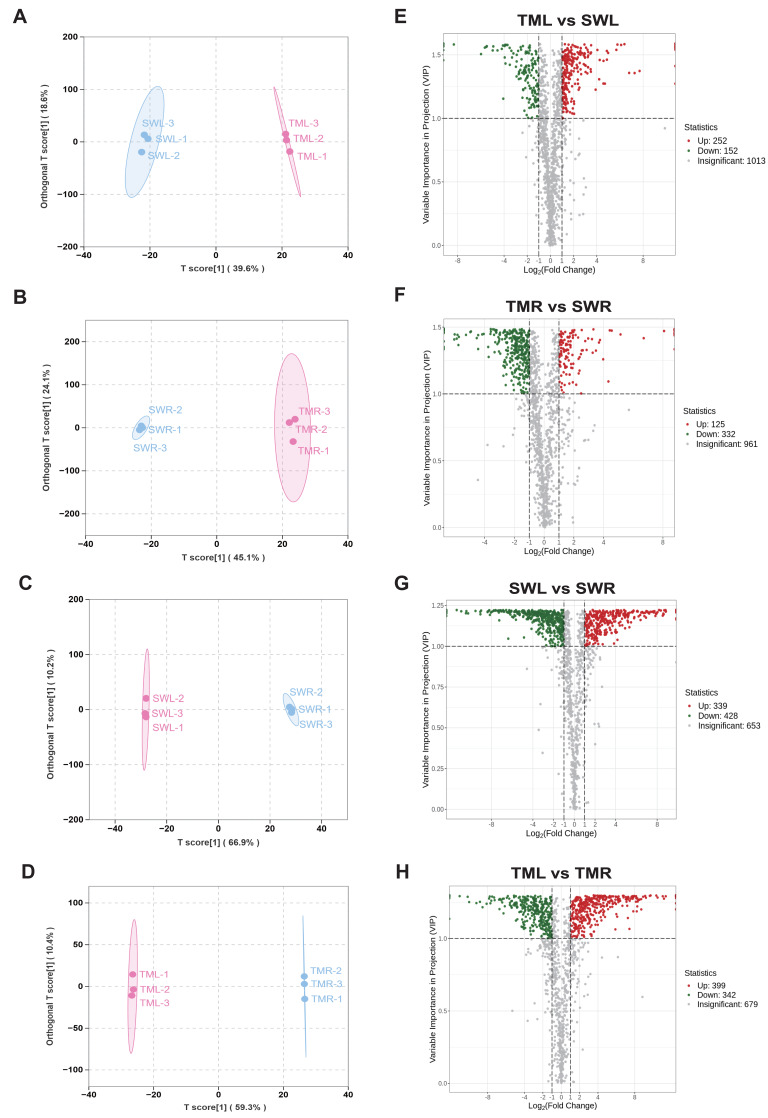
Orthogonal partial least squares discriminant analysis (OPLS-DA) of the organ metabolite difference of each pairwise comparison (A–D). The volcanic maps indicated their corresponding number of significant different metabolites (E–H). The groups of the same organs in different collection sites were represented by SWL *vs* TML and SWR *vs* TMR; the groups of the same collection site in different organs were represented by SWL *vs* SWR and TML *vs* TMR, respectively. The red circles represent the up-regulated components. The green circles represent the down-regulated components.

**Figure 4:**
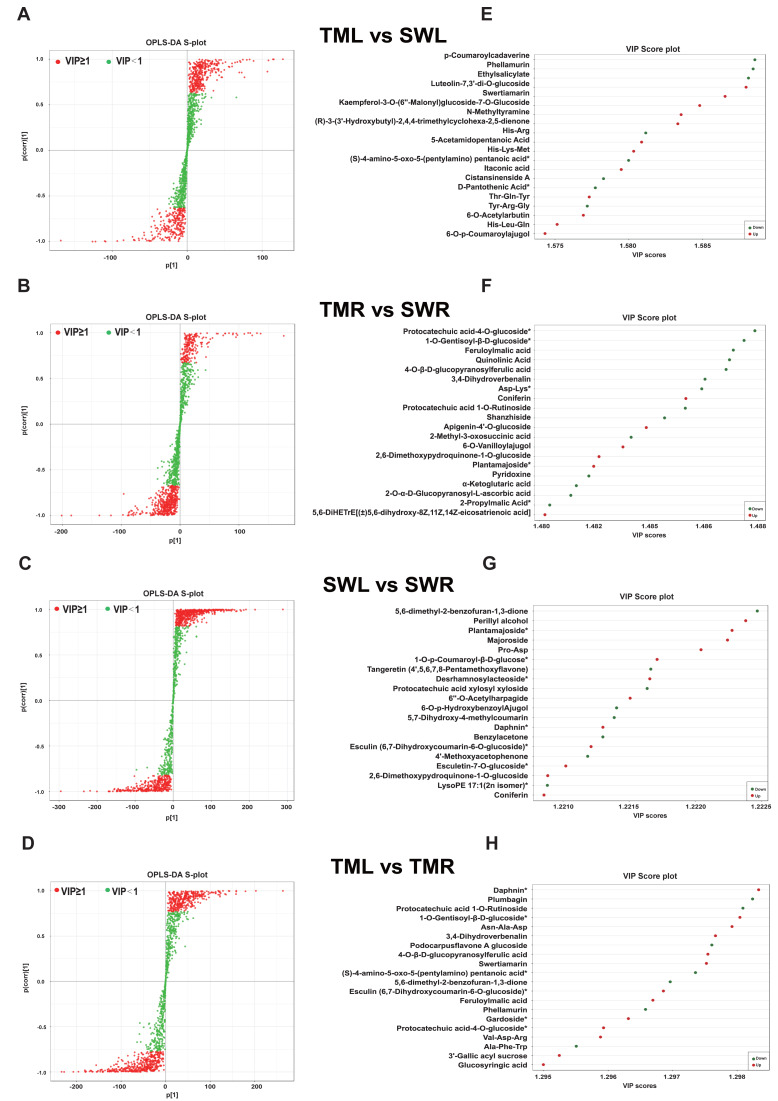
S-plot of OPLS-DA analysis for geographic (A, B) and organ-specific pairwise comparisons (C, D). Their corresponding top 20 compounds with highest VIP scores of all identified metabolites (E–H). Red points indicate metabolites with VIP ≥ 1, while green points indicate metabolites with VIP < 1. Metabolites marked with an asterisk (*) in figures indicate isomers that could not be further distinguished by the current UPLC-MS/MS-based widely targeted metabolomics approach.

To further analyze the differential metabolites in leaves and roots across two distinct growth sites, OPLS-DA was employed for pairwise comparisons of SWL, SWR, TML, and TMR (Figs. 3 and 4). In all pairwise comparisons, the Q^2^ and R^2^Y values exceeding 0.88 for the calculated models demonstrated notable stability and reliability (Fig. S1). Based on the score plots of identified metabolites content, four significant separations were identified among the pairwise comparison groups (Figs. 3A–3D). The corresponding volcano plots revealed that a total of 404 and 457 DSMs existed within the same organ across distinct regions (Figs. 3E, 3F), while notably there were 767 and 741 DSMs between leaves and roots, respectively, of the same sites (Figs. 3G, 3H). These comparisons suggest that the potential accumulation of metabolites in *R. chingii* may be more significantly influenced by the organ development than by regional collection differences.

Further qualitative analysis revealed that the top 20 DSMs in leaves from distinct regions predominantly consisted of six amino acid derivatives: His-Arg, His-Lys-Met, (*S*)-4-amino-5-oxo-5-(pentylamino) pentanoic acid, Thr-Gln-Tyr, Tyr-Arg-Gly, and His-Leu-Gln (Figs. 4A, 4E, Table S2). In contrast, the top 20 DSMs identified in roots were primarily comprised of seven phenolic acids, including protocatechuic acid-4-*o*-glucoside, 1-*o*-gentisoyl-*β*-d-glucoside, feruloylmalic acid, 4-*o*-*β*-d-glucopyranosylferulic acid, coniferin, protocatechuic acid 1-*o*-rutinoside, and plantamajoside (Figs. 4B, 4F, Table S2). Based on the VIP score of OPLS-DA analysis, *p*-coumaroylcadaverine and protocatechuic acid-4-*o*-glucoside could be biomarkers for two geographical regions in *R. chingii*. Additionally, the DSMs obtained from leaves and roots collected in Songwan Village predominantly included five terpenoids and five phenolic acids (Figs. 4C, 4G, Table S2). Similarly, in samples from Tianmu Mountain, seven phenolic acids, four terpenoids, and four amino acid derivatives were detected in the two organs (Table S2). Specifically, 5, 6-dimethyl-2-benzofuran-1, 3-dione, classified as a sesquiterpenoid, and daphnin, a type of coumarin, demonstrated significant accumulation differences between leaves and roots. These compounds were also identified as biomarkers for various organ types in *R. chingii* (Figs. 4D, 4H).

### KEGG annotation of metabolites

**Figure 5:**
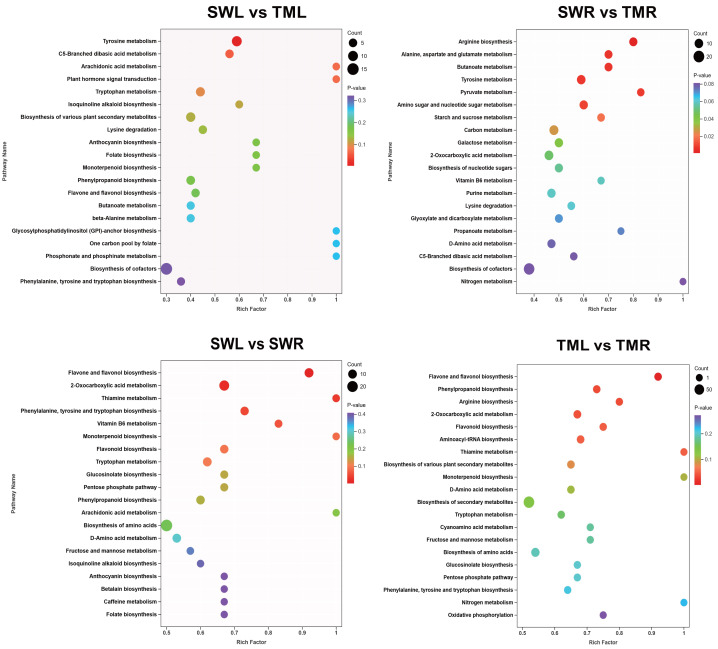
Kyoto Encyclopedia of Genes and Genomes (KEGG) pathways annotations and enrichment analysis of four pairwise comparison groups from two *R. chingii* organ-specific metabolites. Circles of different colors represent different degrees of correlation of *p*-values, and the size represents the number of enriched pathways.

The KEGG pathway enrichment was utilized to analyze the potential biosynthesis features of the DSMs (Kanehisa & Goto, 2000). Figure 5 illustrates the top 20 pathways identified through KEGG enrichment analysis. In a comparison group of identical organs from different regions of *R. chingii*, tyrosine metabolism showed the most significant enrichment pathway and the largest number (10) in the leaves (*p* < 0.01) (Fig. 5A). Additionally, six significantly enriched pathways were found in the roots, mainly including arginine biosynthesis (eight), alanine, aspartate and glutamate metabolism (seven), butanoate metabolism (seven), tyrosine metabolism (10), pyruvate metabolism (five), and amino sugar and nucleotide sugar metabolism (nine) (*p* < 0.01) (Fig. 5B). In different organs of the same *R. chingii* plant, the most significantly enriched pathways in the SWL *vs* SWR and TML *vs* TMR groups were both the flavone and flavonol biosynthesis pathways (11) (*p* < 0.01) (Figs. 5C, 5D).

### Identification of the pharmaceutical ingredients based on the TCMSP database

**Figure 6:**
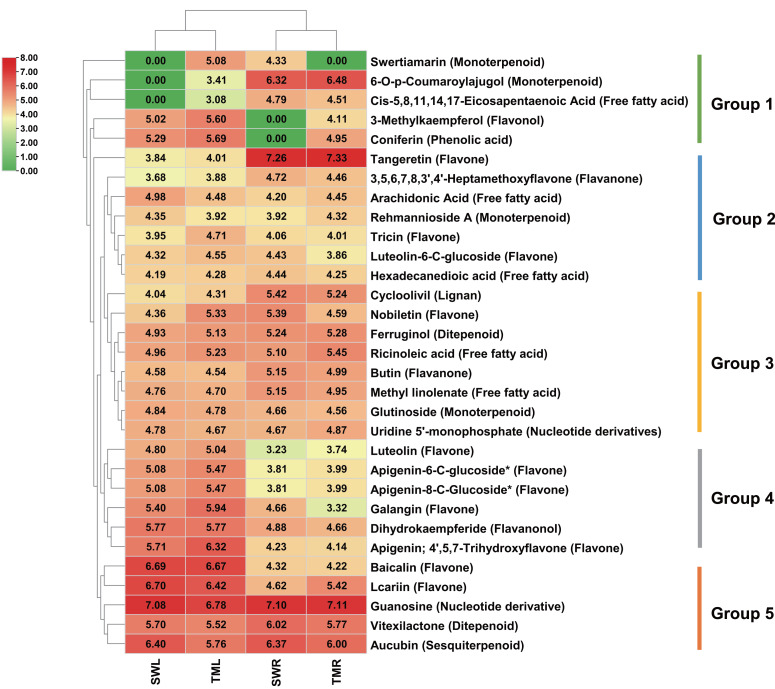
The relative content comparison heatmap in different *R. chingii* organs combined with potential medicinal ingredients screened in TCMSP database. The selected ingredient thresholds were set at oral bioavailability (OB) ≥30% and drug similarity (DL) value ≥0.18. The average content values of three biological replicates were processed with the logarithmic basis of 10. They are shown from red to green squares, which indicated the highest to lowest levels.

*R. chingii* are widely recognized as prominent Chinese herbs due to their abundant medicinal properties (Liu et al., 2016; Wang et al., 2025b). Hence, with references to the bioactive functions of *R. glutinosa* (Chen et al., 2021; Zhou et al., 2018), all identified metabolites of *R. chingii* were further screened in the TCMSP database to determine the potential pharmaceutical ingredients in the two organs from different origin samples (Ru et al., 2014). The results indicated that 212 metabolic compositions were identified as traditional Chinese medicine ingredients based on their sufficient OB and DL values in the database (Table S3). To more precisely pinpoint the significant bioactive components, the search thresholds were raised to OB ≥ 30% and DL ≥ 0.18 during this process (Ru et al., 2014). A total of 31 metabolites were identified as key bioactive ingredients with potential efficacy (Fig. 6). Qualitatively, these metabolites are mainly composed of six categories: 15 flavonoids (11 flavones, two flavonols, one flavanone, and one flavanonol), seven terpenoids (four monoterpenoids, one sesquiterpenoid, and two diterpenoids), five lipids, two nucleotide derivatives, one phenolic acid compound, and one lignan compound. Excluding the documented medicinal ingredients (Chen et al., 2021), such as coniferin (a phenolic acid), guanosine and uridine 5’-monophosphate (nucleotide derivatives), cycloolivil (a lignan), and five free fatty acids (lipids), there are other significant bioactive compositions that are crucial for the medicinal functions of *R. chingii*.

Furthermore, a clustering heatmap was used to compare the accumulation levels of significant medicinal ingredients across different organs and growth regions, which revealed that two groups were distinct in SWL, TML, and SWR, TMR (Fig. 6). This finding was partly consistent with the separation of leaves and root metabolites in Fig. 1C, which further indicates that organ-specific distribution of pharmaceutical ingredients exists in the distinct regions. Additionally, all ingredients were classified into five groups based on their unique content distribution. In Group 1, swertiamarin (an iridoid glycoside) was predominantly detected in TML and SWR. Furthermore, 6-*o*-*p*-coumaroylajugol (a monoterpenoid), *cis*-5, 8, 11, 14, 17-eicosapentaenoic acid (a free fatty acid), 3-methylkaempferol (a flavonol), and coniferin (a phenolic acid) were nearly undetectable in SWL and SWR.

The highest levels of the medicinal metabolite, tangeretin (a flavone) from Group 2, were observed in the *R. chingii* roots growing in both distinct regions. Most medicinal components in Groups 3 and 4 exhibited higher accumulation in the roots and leaves, respectively. Additionally, multiple flavonoids clustered in Group 4 were predominantly distributed in *R. chingii* leaves. Moreover, multiple medicinal components in Group 5 also exhibited high content, particularly guanosine (a nucleotide derivative), which was found in elevated levels in both roots and leaves.

### Comparative analysis of pharmaceutical ingredients for predicting the treatment of five diseases

**Figure 7:**
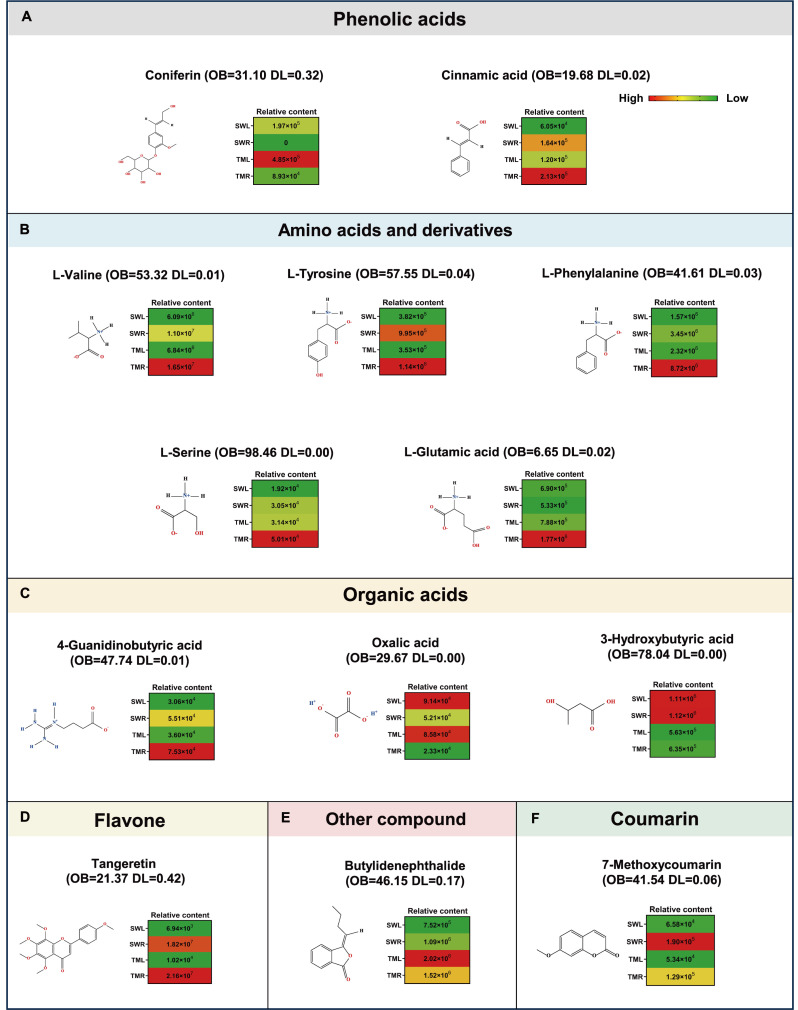
Comparison of key medicinal ingredient contents across collection sites and organs. As retrieved from the TCMSP database, these metabolites could collectively be involved in the therapeutic applications of *R. chingii*, which include treatment of cancer/tumor, cardiovascular disease, Alzheimer’s disease, diabetes mellitus, and depression. Six categories of potential medicinal ingredient structures and two key network pharmacological indices (OB and DL values) for traditional Chinese medicine preparations were displayed, separately. Green to red indicate the highest to lowest content.

It has been previously reported that the primary medicinal properties of the *Rehmannia* genus mainly include anti-cancer/tumor, anti-cardiovascular disease, anti-diabetic, and anti-depressant effects, as well as benefits in the treatment of Alzheimer’s disease (Chen et al., 2021; Liu et al., 2017). In conjunction with the search results from the TCMSP database, we further investigated the individual compounds that may contribute to these five medicinal functions in *R. chingii*. The results indicated that six categories of pharmaceutical ingredients could play roles in bioactive functions, including coniferin and cinnamic acid (a phenolic acid), five amino acids and their derivatives, three organic acid compounds, tangeretin (a flavone), butylidenephthalide (an unclassified compound), and 7-methoxycoumarin (a coumarin) (Fig. 7). Additionally, the organ-specific accumulation in two regions was also analyzed using clustering heatmaps. Notably, the contents of the two identified phenolic acids and five amino acid derivatives, 4-guanidinobutyric acid, tangeretin, and butylidenephthalide were found to be higher in TML and TMR compared to SWR and SWL (Figs. 7A, 7B). Conversely, the accumulation levels of oxalic acid, 3-hydroxybutyric acid, and 7-methoxycoumarin were greater in SWR and SWL (Figs. 7C, 7F).

## Discussion

In this study, we comprehensively characterized the complex and diverse profiles of metabolites in the leaves and roots of *R. chingii* from two distinct regions. Compared with *R. glutinosa*, which has been extensively studied for its diverse compositions (Zhou et al., 2016; Feng et al., 2015), our findings revealed a higher abundance of phenolic acids, amino acid derivatives, and lipid compounds (Fig. 1D). This highlights the intricate metabolic diversity within the *Rehmannia* genus and suggests that *R. chingii* may possess unique bioactive properties. Furthermore, the diversity of identified compounds can be partially explained to the use of methanol as the extraction solvent in our pre-treatment method. It is known for its efficiency in capturing a wide range of polar and non-polar compounds, thereby facilitating the detection of a broader spectrum of metabolites (Jha & Sit, 2022). Moreover, the variations in mass spectrometry detection parameters employed in our study further contributed to the qualitative diversity of the identified metabolites (Wang et al., 2021). Compared to previous studies on the analysis of specific iridoid glycosides and polysaccharides of *R. glutinosa* (Wang et al., 2020), our detailed characterization of the metabolite profiles in *R. chingii* offers a more holistic view of its phytochemical composition. This information could be valuable for fully elucidating the bioactive potential of the *Rehmannia* genus.

The geo-herbalism framework posits that the efficacy of medicinal plants is intrinsically linked to their geographical origins (Wang et al., 2018). This concept is further substantiated by evidence indicating that variations in environmental factors can significantly influence the types and concentrations of metabolites (Li et al., 2020). In our study, the accumulation levels of detected metabolites in *R. chingii* are distinctly influenced by its geographical regions (Figs. 1C, 3A, 3B, 4A, 4B), thereby underscoring the importance of considering the geographical distribution of specific ingredients for evaluating the medicinal potential in *R. chingii* and related species (Tian et al., 2023). Notably, the marked differences in root metabolites between Tianmu Mountain and Songwan Village likely reflect adaptive responses to contrasting environmental conditions (Fig. 1C). Specifically, a greater number of phenolic acids and their individual components (coniferin and cinnamic acid) show enhanced accumulation in *R. chingii* from Tianmu Mountain (Figs. 1D, 7A). This pattern may be attributed to the montane forest habitats that exhibited higher altitude and associated environmental stresses, including potentially cooler temperatures and stronger UV radiation. Moreover, phenolic acids are well-documented to play critical roles in abiotic stress adaptation, such as scavenging reactive oxygen species or reinforcing cell wall integrity to withstand harsh conditions (Naikoo et al., 2019; Kumar et al., 2023). Additionally, researches have shown that cinnamic acid and its derivatives possess a broad spectrum of pharmacological properties, including anti-cancer, antibacterial, anti-inflammatory, antidepressant, and blood sugar-lowering effects (Tian et al., 2025b). Similarly, the coniferin and cinnamic acid we identified were also found in the TCMSP database as key components potentially contributing to five important biological activities of the genus *Rehmannia*. Thus, we infer that phenolic acids may serve as the key medicinal marker components by which *R. chingii* responds to natural environmental factors. Furthermore, KEGG enrichment analysis revealed significant variations in flavone and flavonol biosynthesis between leaves and roots (Figs. 5C, 5D). This distinction between phenotypic traits and pathway enrichment highlights the modular nature of metabolic system in *R. chingii*: flavonoid biosynthesis is predominantly enriched in the flavonoid pathway (ko00941), whereas phenolic acids are involved in multiple discrete pathways (Yu et al., 2020; Kanehisa & Goto, 2000; Ma et al., 2015). In future research, we will further explore the underlying genetic and environmental mechanisms driving these metabolic patterns, and this is essential for understanding their significance in targeted therapeutic applications of *R. chingii* and related species.

The differential classification and accumulation of organ-specific bioactive ingredients represent a critical factor influencing the efficacy of TCM, as these variations directly determine the material basis of therapeutic effects (Ru et al., 2014). In our study, TCMSP database identified distinct organ-specific accumulation patterns of potential medicinal flavonoids in *R. chingii* (Fig. 6). In particular, baicalin and luteolin, both belonging to the flavone class, were predominantly accumulated in the leaves, whereas tangeretin, a flavone C-glycoside, exhibited the highest concentration in the roots (Fig. 7D). This organ-specific distribution aligns with the well-documented interorgan biosynthesis specificity of flavonoids in plants, where leaves, as primary photosynthetic organs, often synthesize flavonoids for light protection and defense (Falcone Ferreyra, Rius & Casati, 2012). As storage and secondary metabolic hubs, roots may accumulate specific flavonoids, especially in tangeretin (Fig. 7D), to fulfill unique physiological or ecological roles (Liu et al., 2021). Besides, baicalin and luteolin are the well-characterized natural antimicrobial compound with broad-spectrum activity against bacteria, fungi, and viruses (Ozma et al., 2021; Mahamud et al., 2024). And tangeretin is known for its neuroprotective functions and anticancer effects against colorectal and lung cancers (Fatima & Siddique, 2025; Zhao et al., 2023a). Our investigation revealed that the roots of *R. chingii* sourced from the Tianmu Mountains are a critical reservoir of tangeretin implicated in the treatment of five intractable diseases (Fig. 7D; Table S4). The favorable OB and DL values indicate that it may serve as a promising candidate for future research into the molecular synthesis and regulation of key medicinal metabolites in *R. chingii*. Therefore, in addition to the well-studied iridoids and their derivatives, such as catalpol, rehmannioside, and verbascoside (Liu et al., 2016; Chen et al., 2021; Wang et al., 2025b), the organ-specific of flavonoids in *R. chingii* warrant further investigation, which could provide valuable insights into the medicinal potential of *Rehmannia* species and contribute to the development of targeted therapeutic applications.

Additionally, in an arable field edge-like habitat in Rui’an, Wenzhou, the accumulation levels of two organic acids in *R. chingii* were observed. Oxalic acid was predominantly enriched in the leaves, while 3-hydroxybutyric acid accumulated to significantly higher levels in the roots (Fig. 7C). This phenomenon suggests that the microhabitat conditions of southern regions may regulate metabolic pathway, influencing the synthesis and distribution of specific organic acids. These regions may receive higher light intensity due to sparse surrounding crops, experience more frequent water management, or have richer soil nutrient inputs. Such conditions could alter the tricarboxylic acid cycle and photosynthetic mechanisms, thereby affecting organic acid accumulation (de Oliveira et al., 2025). From a medicinal perspective, oxalic acid could reduce calcium accumulation and prevent kidney stone formation (Chen et al., 2024). Moreover, 3-hydroxybutyric acid involved in the regulation of energy metabolism, which has the potential to treat neurological diseases due to its neuroprotective properties (Laeger, Metges & Kuhla, 2010). These findings further expand the understanding of metabolic diversity in *R. chingii*: beyond the known flavonoids, phenolic acids, and iridoid components, differences in organic acid accumulation across habitats may also represent an important mechanism by which *R. chingii* adapts to its environment and exerts specific medicinal functions. Future studies could integrate multi-omics data to elucidate the molecular mechanisms underlying synthesis of secondary metabolites, providing a more comprehensive theoretical basis for the development of medicinal resources in *R. chingii*.

It is also noteworthy that multiple primary metabolites, particularly five isolated amino acid derivatives, are likely involved in the documented bioactivities of the *Rehmannia* genus, as indicated by the search of the TCMSP database (Ru et al., 2014). However, these compounds exhibited higher oral bioavailability (OB) values (6.65–98.46) but lower drug-likeness (DL) values (0–0.04), suggesting a significant discrepancy in the chemical space between natural products and synthetic drugs (Fig. 7B, Table S4). This phenomenon may be related to the presence of polar functional groups (such as amino and carboxyl groups) in their structure. Although polar groups promote intestinal absorption through active transport mechanisms, they simultaneously lead to an imbalance in the lipid-water partition coefficient, which does not satisfy the lipid solubility requirements of traditional drug-like molecules (Lipinski et al., 1997). As primary metabolites, these components may exert their medicinal effects by regulating the amino acid metabolism of the host rather than relying on the binding of a single target (Zhang et al., 2020). While the drug-likeness criteria established by TCMSP are widely applied to chemically synthesized compounds, their predictive validity for natural products, including the identified bioactive components in *R. chingii*, remains unvalidated concerning *in vivo* therapeutic potential, particularly in relation to their proposed roles in treating the five diseases mentioned. This limitation highlights the necessity for further experimental verification, including *in vivo* pharmacodynamic studies, pharmacokinetic evaluations, and target engagement assays, to confirm their therapeutic potential and elucidate the underlying mechanisms. Such investigations will not only validate the speculative disease-related associations but also provide crucial insights into optimizing the medicinal application of *R. chingii* by establishing structure–activity relationships and ensuring the reliability of its proposed therapeutic efficacy.

To address the practical implications for the harvesting, processing, and medicinal use of *R. chingii*, our findings underscore the importance of organ-specific accumulation of bioactive compounds and the geographical variability in their composition. Specifically, when utilizing this medicinal plant, it is crucial to prioritize geographical origin due to the distinct metabolic profiles observed between the two regions, which suggest the existence of region-specific bioactive signatures that necessitate regional quality control and organ specificity. These insights can inform optimized cultivation practices for harvesting and processing, including the selection of the most metabolically productive regions and the adjustment of growth conditions to enhance target metabolite accumulation. Additionally, the implementation of post-harvest strategies is essential to prevent the degradation of organ-specific compounds. Ultimately, integrating geographical origin and organ-specific composition into resource management will ensure the rational use of *R. chingii* and provide a theoretical basis for developing standardized, region-specific Traditional Chinese Medicine formulations.

## Conclusion

Widely targeted metabolomics revealed significant differences in 1,420 secondary metabolites between the leaves and roots of *R. chingii* across two geographical locations. Among the 31 potential medicinal ingredients identified through TCMSP, flavonoids were found to be highly abundant, with enriched biosynthesis pathways noted in KEGG. Notably, coniferin (leaves) and tangeretin (roots) that selected based on their higher oral bioavailability (OB) and drug-likeness (DL) values in TCMSP accumulated to significantly higher levels in samples from Tianmu Mountain. This suggests that they are key candidates for the medicinal properties of *R. chingii*. Overall, this study enhances our understanding of the metabolomic composition of *R. chingii* and uncovers the accumulation patterns of potential medicinal ingredients influenced by geography and organ specificity, thereby providing a foundational dataset for its therapeutic exploration.

##  Supplemental Information

10.7717/peerj.20722/supp-1 Supplemental Information 1 OPLS-DA verification diagram of each pairwise comparisons. X-axis represent the correlation model R 2 Y (orange colors) and Q 2 (purple colors) values. The Y-axis is the frequency of the classification effect in the random permutation combination exper

10.7717/peerj.20722/supp-2 Supplemental Information 2 Overlaid diagram of the total ion current (TIC) from mass spectrometry analysis of quality control samples. (a: negative ion mode; b: positive ion mode)

10.7717/peerj.20722/supp-3 Supplemental Information 3 TIC overlap chromatogram of mass spectrometry analysis of mixed samples ( a: negative ion mode; b: positive ion mode )

10.7717/peerj.20722/supp-4 Supplemental Information 4 The coefficient of variation (CV) distribution diagram for four samples is presented The horizontal axis denotes the CV values, while the vertical axis illustrates the proportion of substances with CV values less than the corresponding value relative to the total number of substances. Different colors represent distinct sample groups. The two reference lines perpendicular to the X-axis correspond to CV values of 0.3 and 0.5, while the two reference lines parallel to the X-axis indicate 75% and 85% of the total number of substances. The samples are categorized as follows: QC (quality control sample), SWL (leaves from Songwan Village, Wenzhou), SWR (roots from Songwan Village, Wenzhou), TML (leaves from Tianmu Mountain, Lin’an , Hangzhou), and TMR (roots from Tianmu Mountain, Lin’an , Hangzhou).

10.7717/peerj.20722/supp-5 Supplemental Information 5 Chromatogram displaying multiple peaks for the detection of metabolites in multiple reaction monitoring (MRM) mode (a: negative ion mode; b: positive ion mode)

10.7717/peerj.20722/supp-6 Supplemental Information 6 Diagram for integrated correction of quantitative analysis concerning metabolites (15 metabolites selected at random, a: mode of negative ions; b: mode of positive ions)

10.7717/peerj.20722/supp-7 Supplemental Information 7 Reassess the correlation evaluation plot. a. Diagram illustrating the correlation among quality control samples. b. Diagram depicting the correlation between test samples

10.7717/peerj.20722/supp-8 Supplemental Information 8 Supplementary tables of metabolites information

## Additional Information and Declarations

### Data Availability

The following information was supplied regarding data availability: The raw measurements are available in the Supplementary Files.
