# Supplementary figures and images for: Comparative metabolomics reveals organ-specific discrepancy in TCMSP-predicted bioactive ingredients between two geographically distinct regions of Rehmannia chingii
Source: PeerJ. 2026 Feb 3;14:e20722. doi: 10.7717/peerj.20722 (PMC12880092; doi:10.7717/peerj.20722)

A

## TML vs SWL

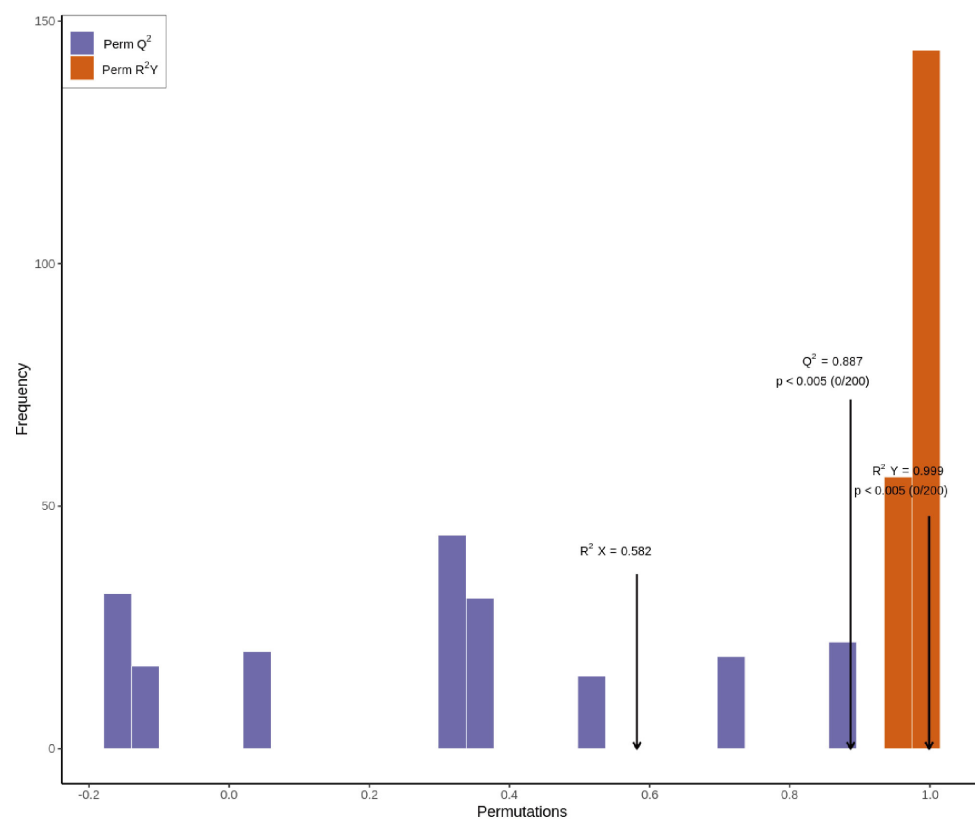

B

## TMR vs SWR

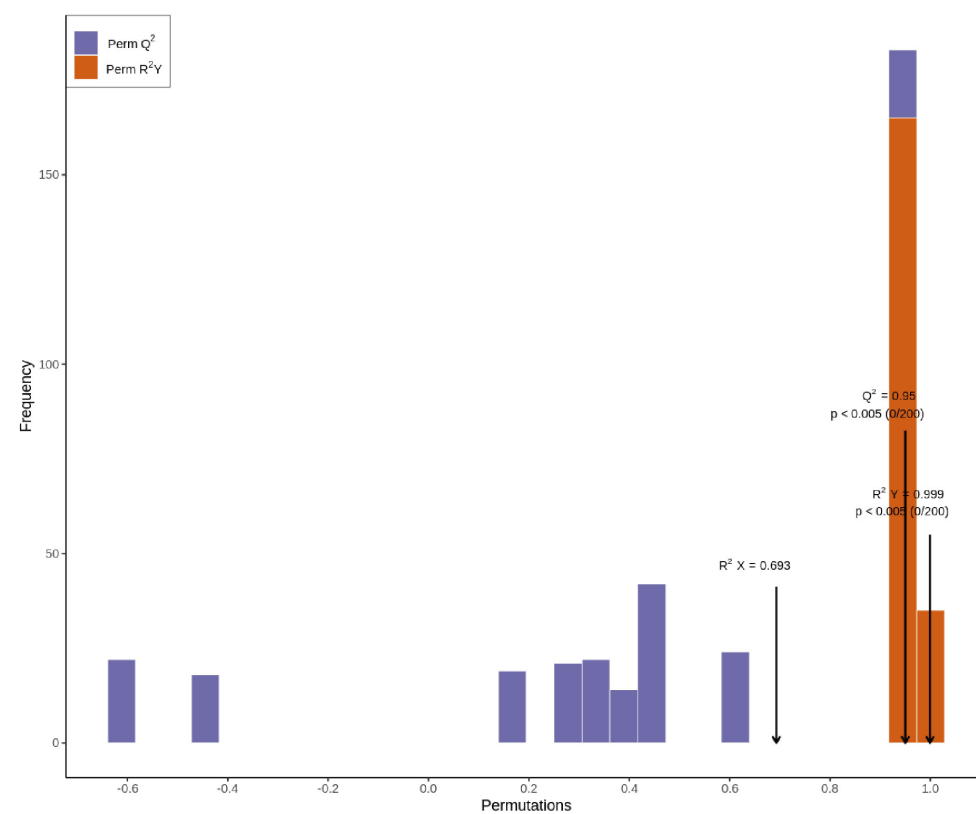

C

## SWL vs SWR

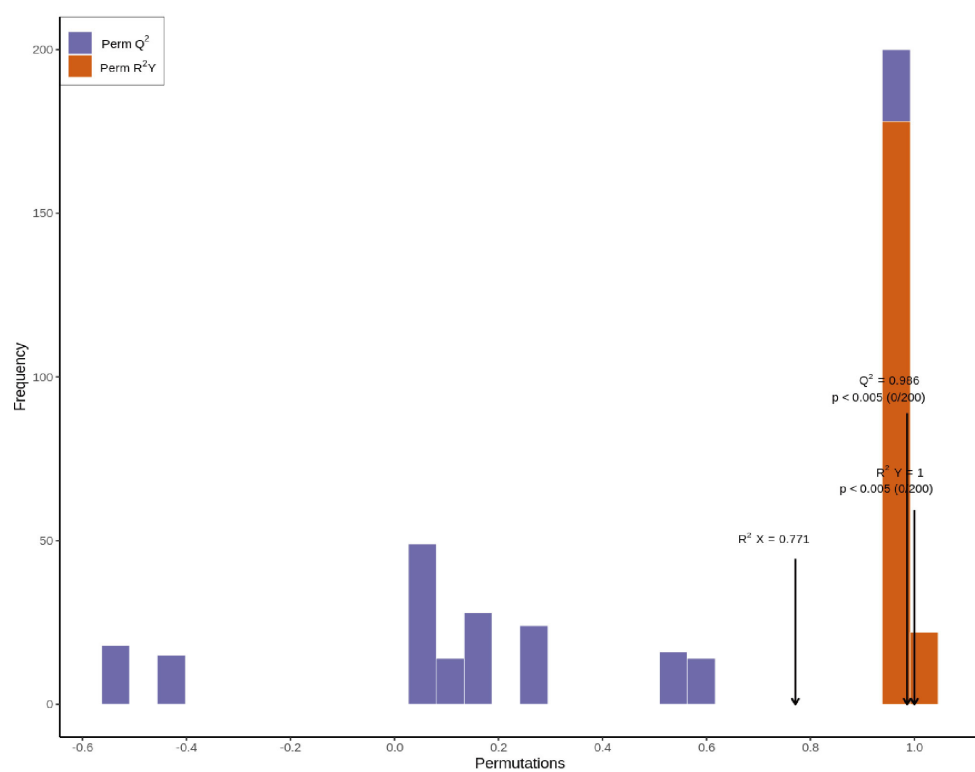

D

## TML vs TMR

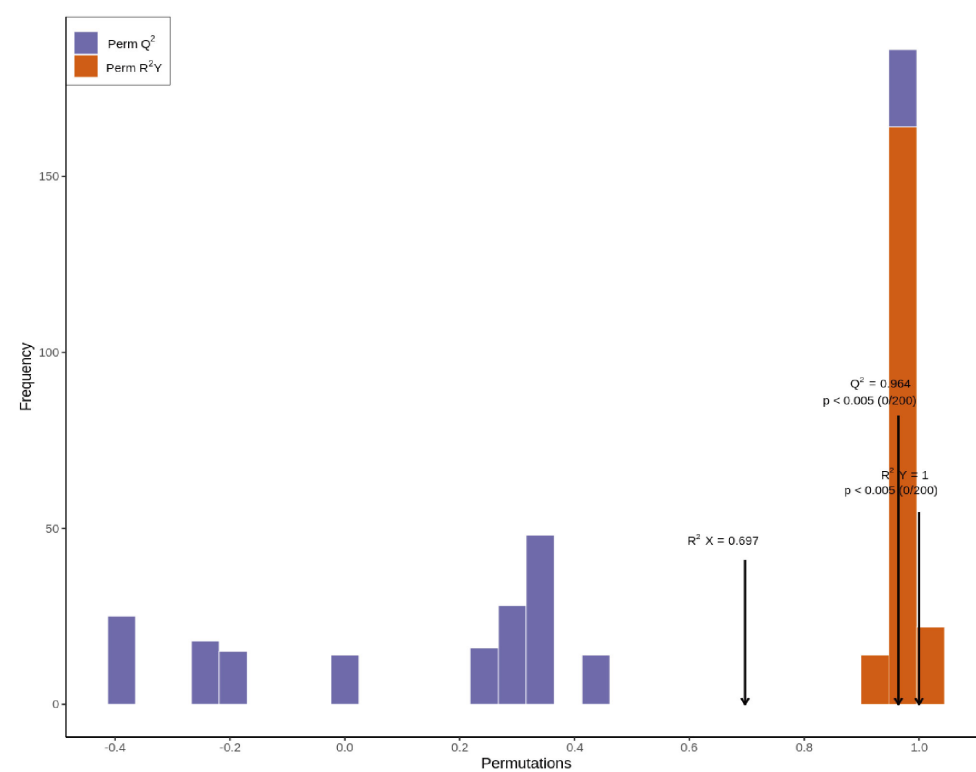

Supplement: Supplemental Information 1 [file peerj-14-20722-s001.pdf]

**A**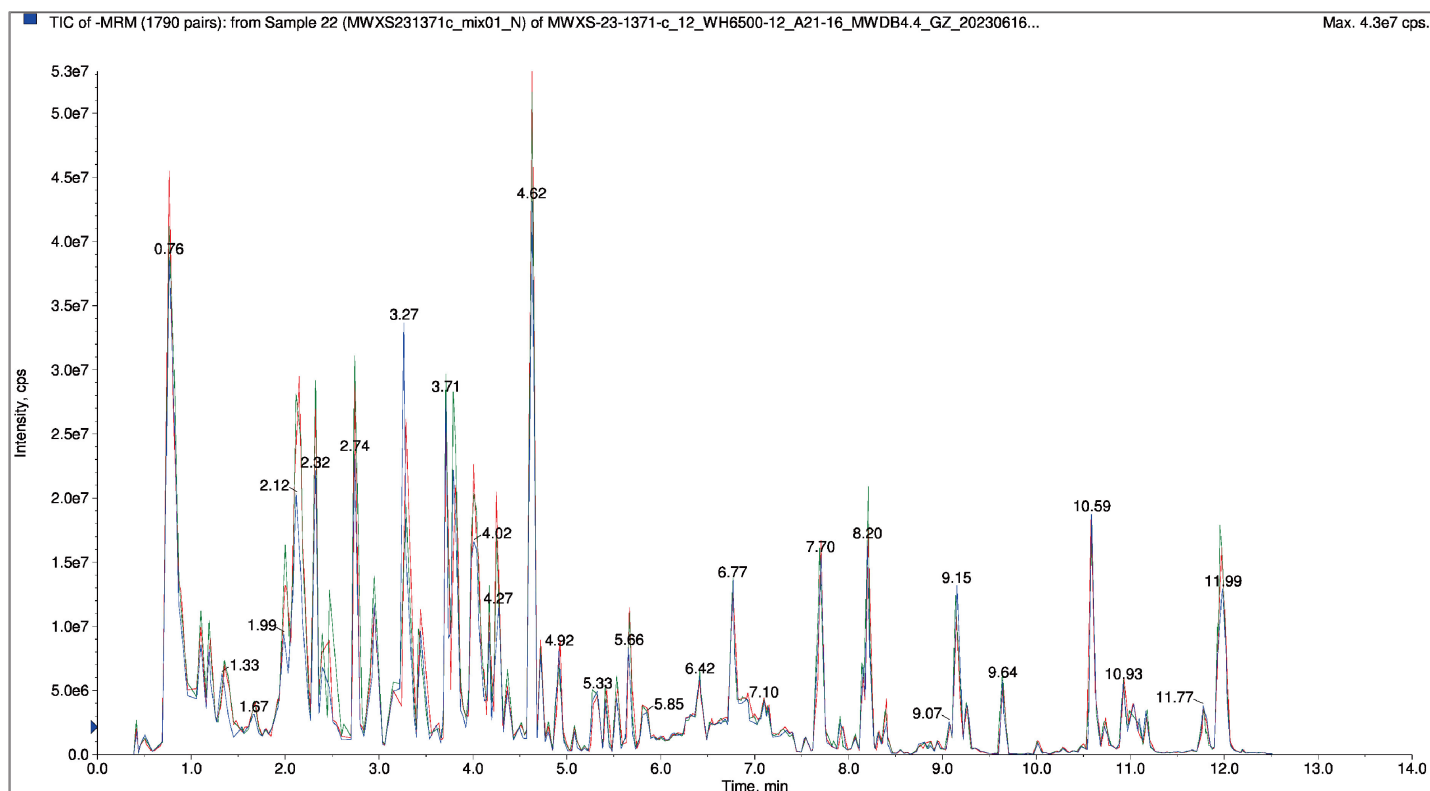**B**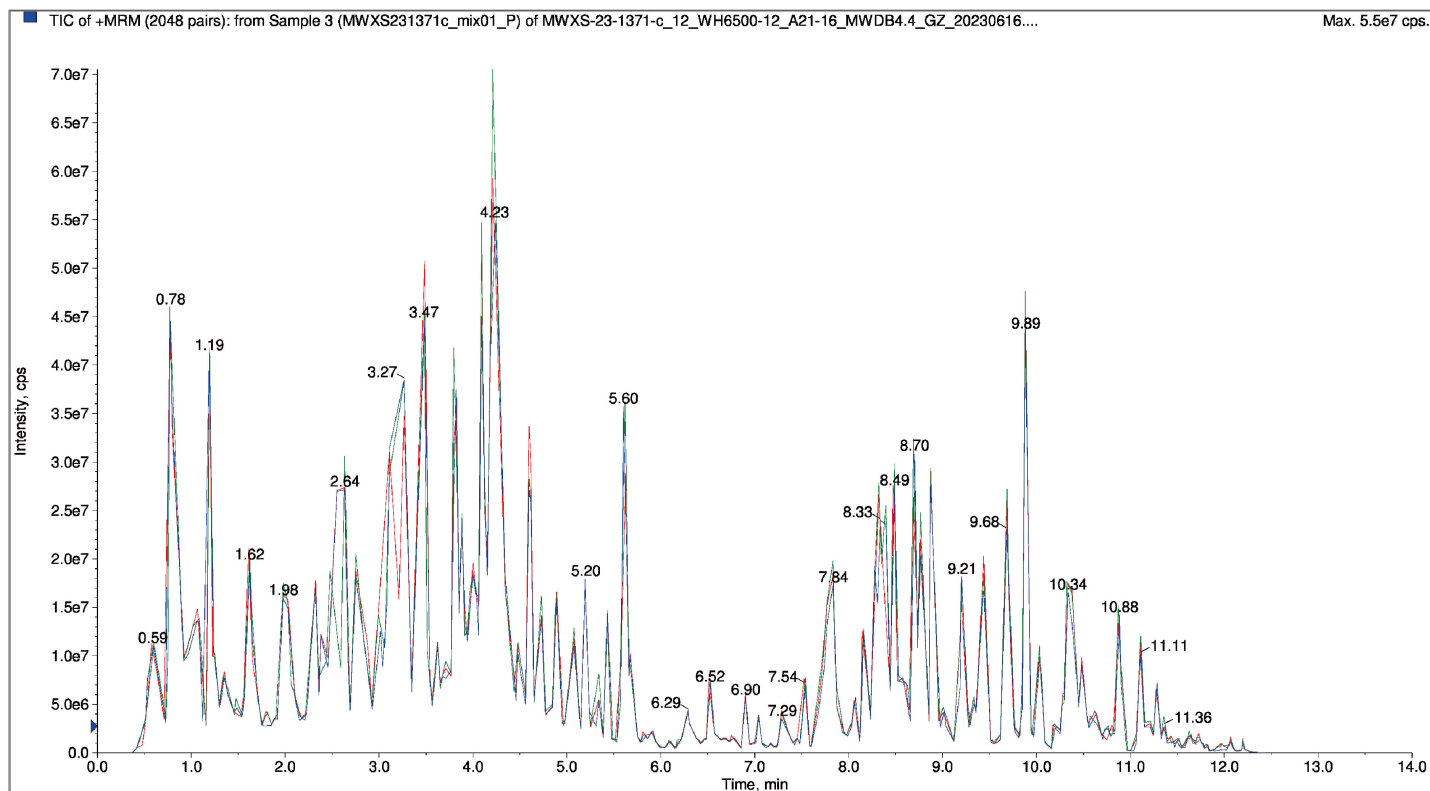

Supplement: Supplemental Information 2 [file peerj-14-20722-s002.pdf]

**A**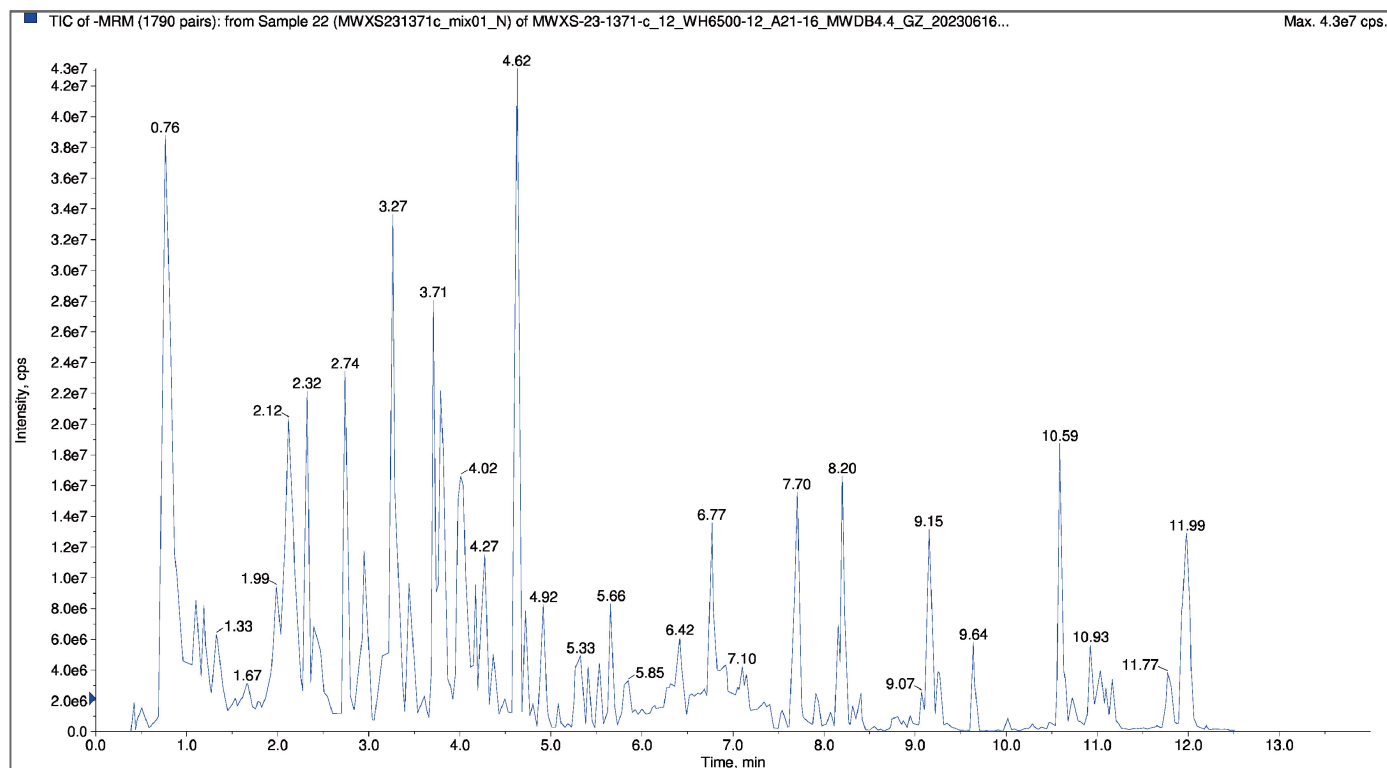**B**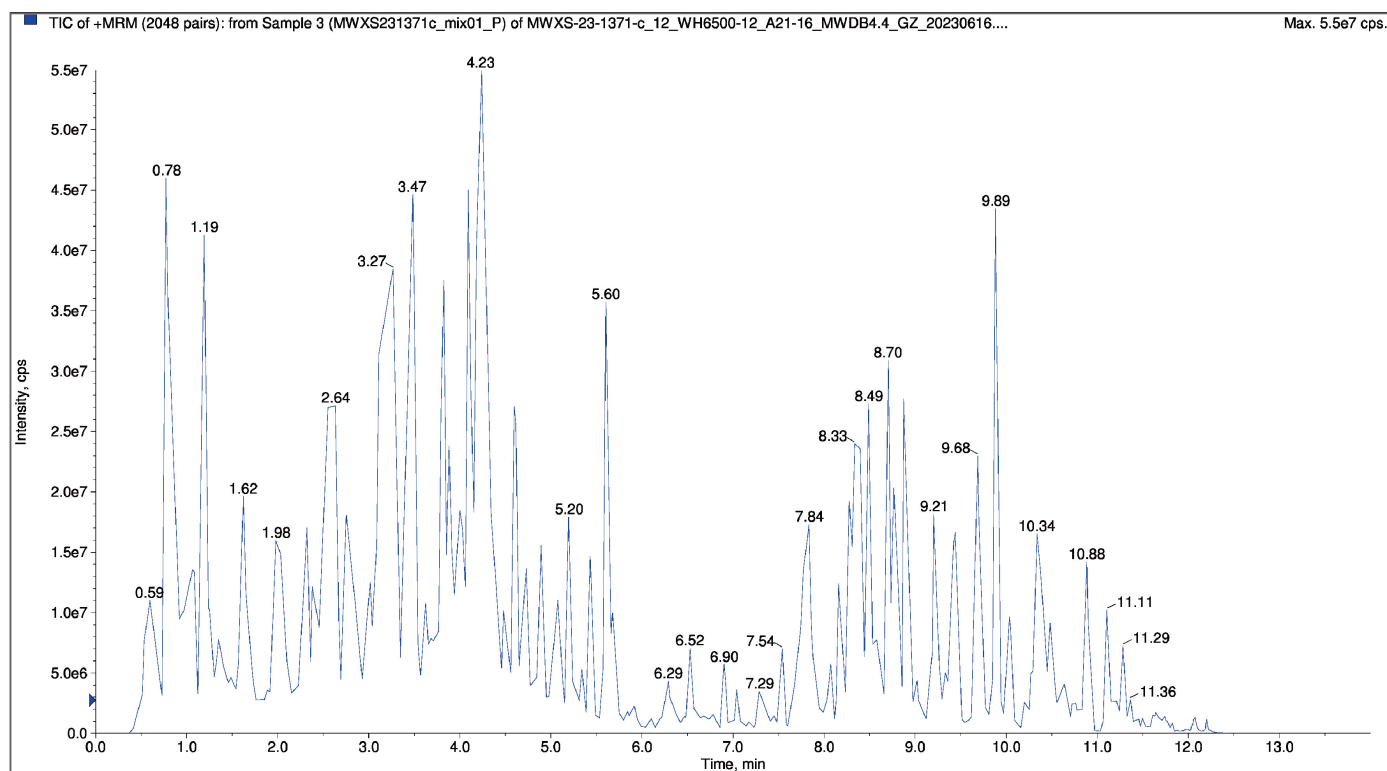

Supplement: Supplemental Information 3 [file peerj-14-20722-s003.pdf]

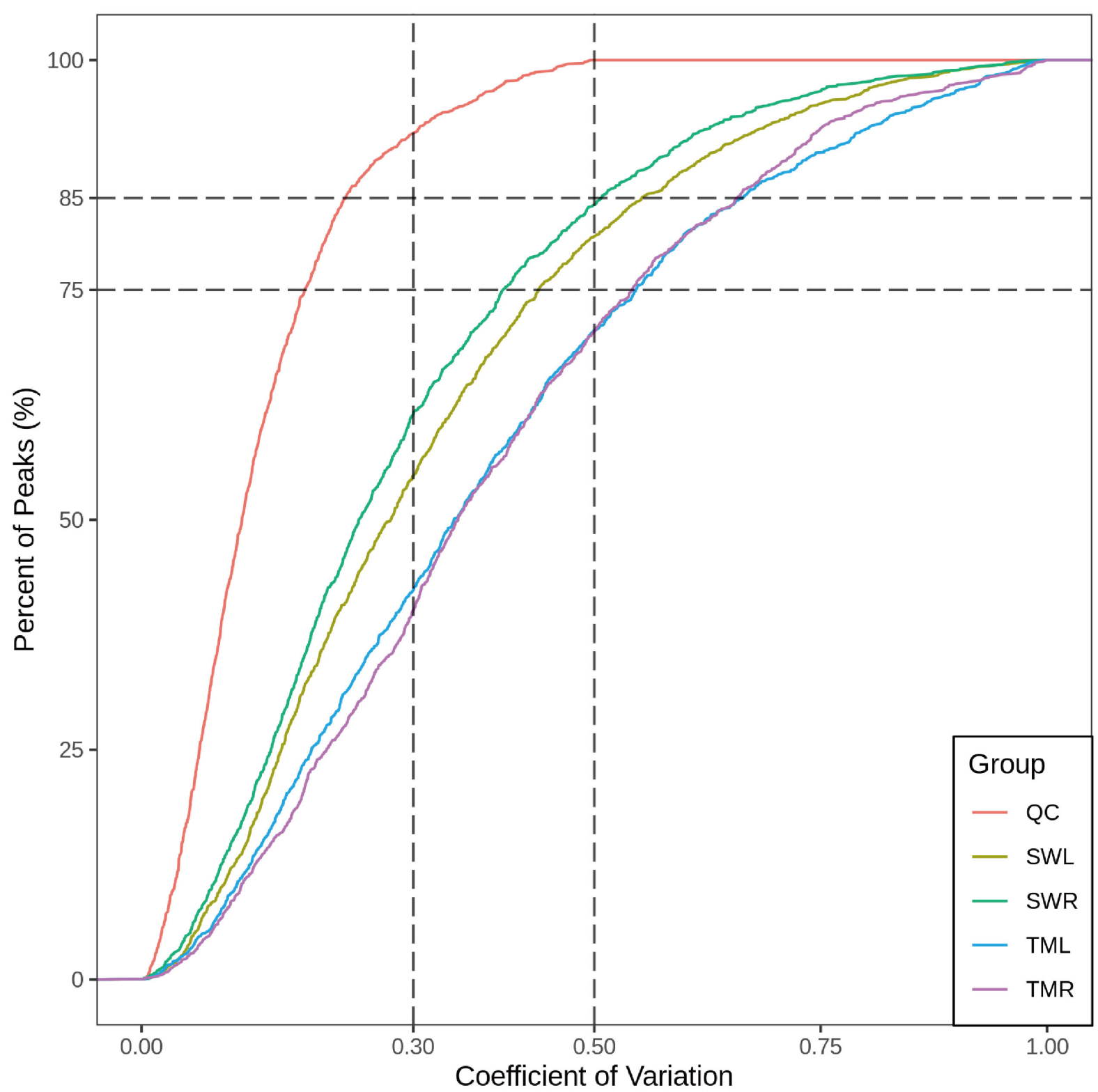

Supplement: Supplemental Information 4 — The horizontal axis denotes the CV values, while the vertical axis illustrates the proportion of substances with CV values less than the corresponding value relative to the total number of substances. Different colors represent distinct sample groups. The two reference lines perpendicular to the X-axis correspond to CV values of 0.3 and 0.5, while the two reference lines parallel to the X-axis indicate 75% and 85% of the total number of substances. The samples are categorized as follows: QC (quality control sample), SWL (leaves from Songwan Village, Wenzhou), SWR (roots from Songwan Village, Wenzhou), TML (leaves from Tianmu Mountain, Lin’an , Hangzhou), and TMR (roots from Tianmu Mountain, Lin’an , Hangzhou). [file peerj-14-20722-s004.pdf]

**A**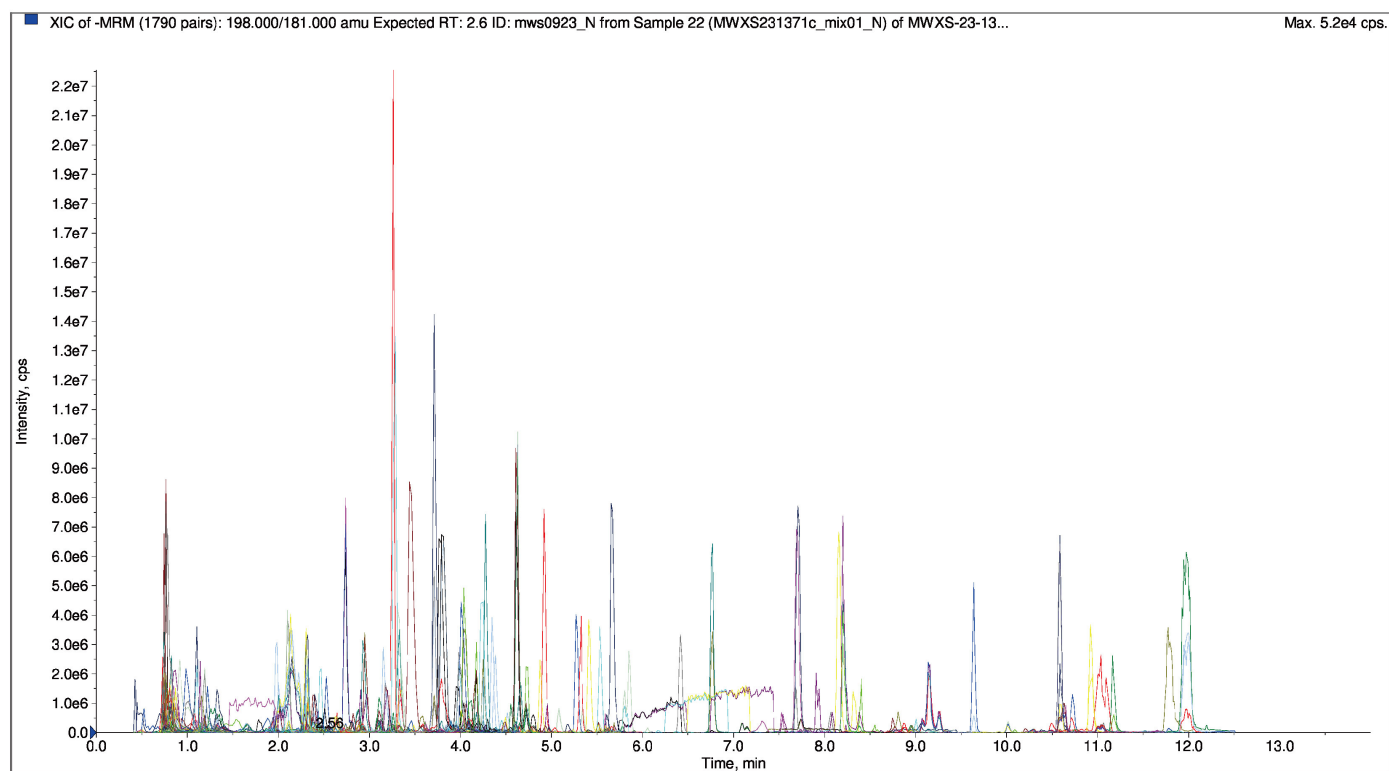**B**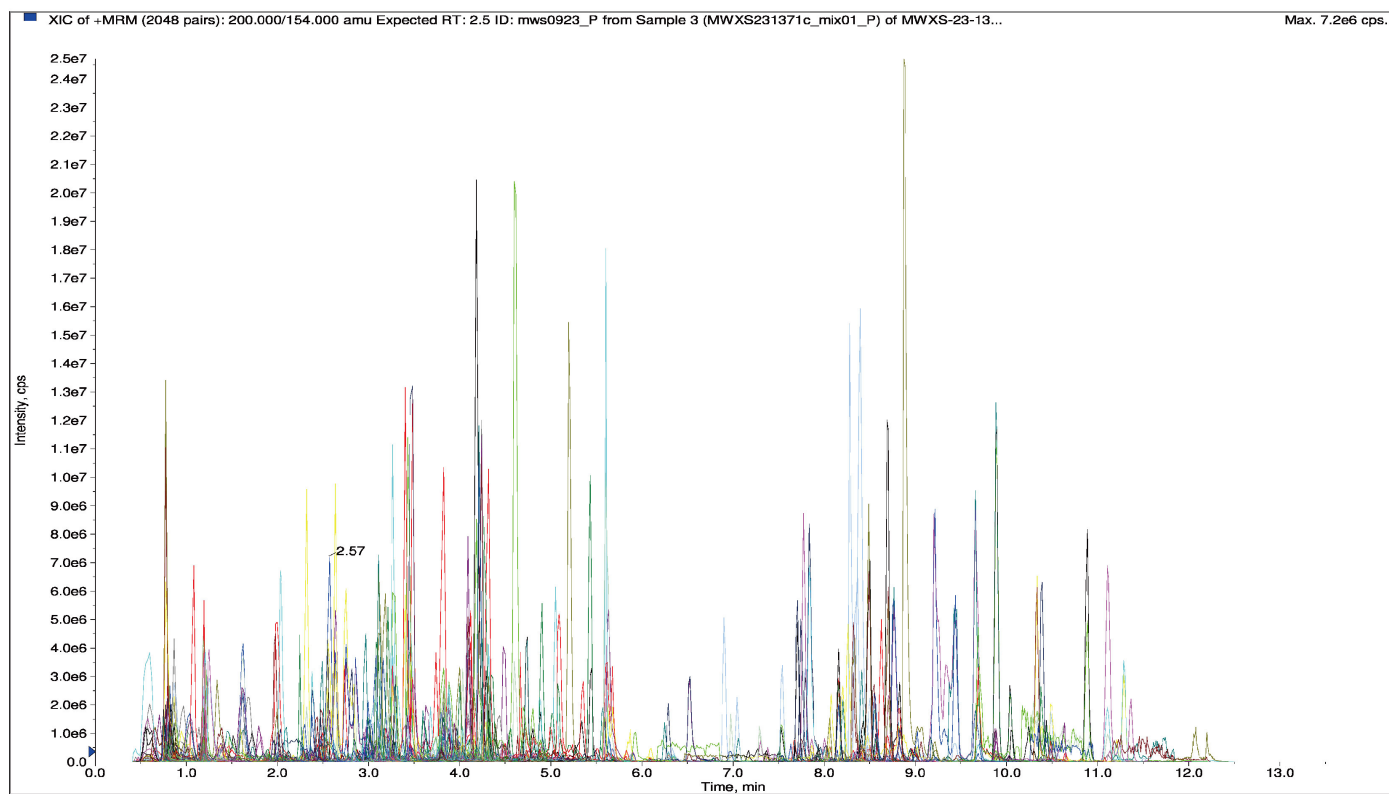

Supplement: Supplemental Information 5 [file peerj-14-20722-s005.pdf]

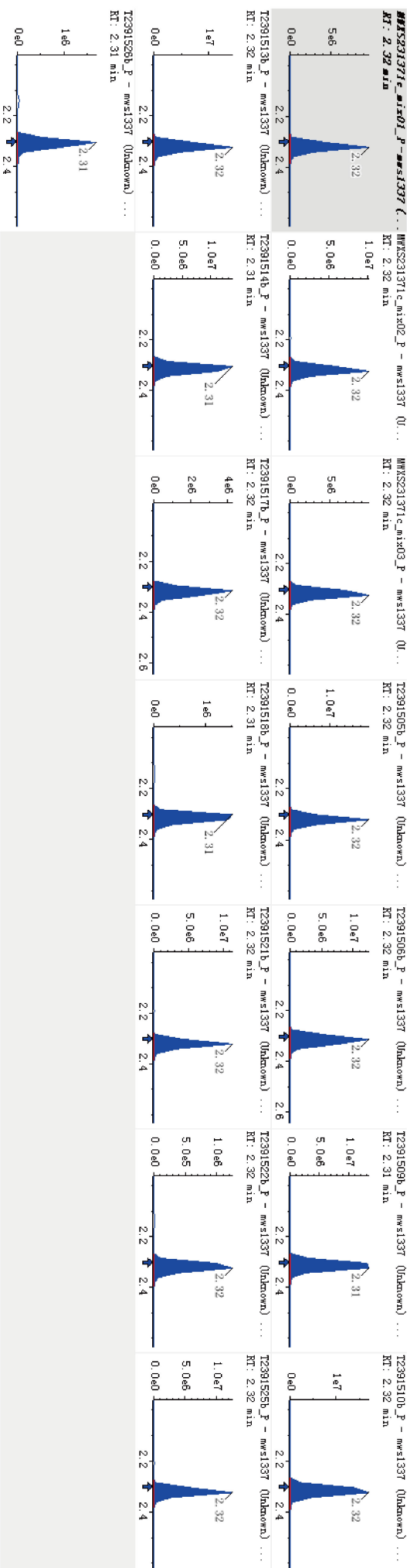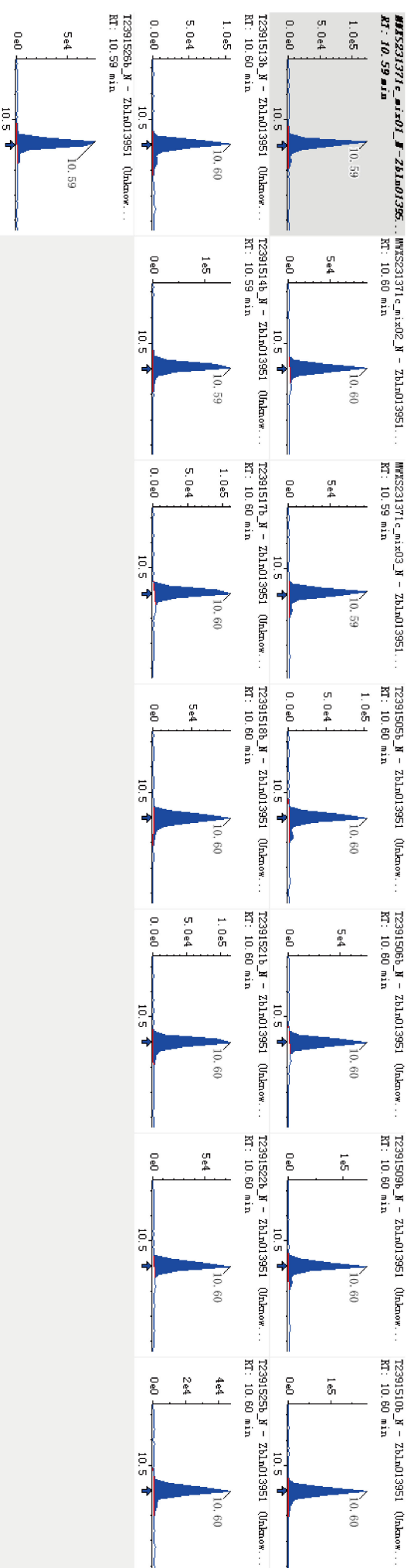

Supplement: Supplemental Information 6 [file peerj-14-20722-s006.pdf]

A

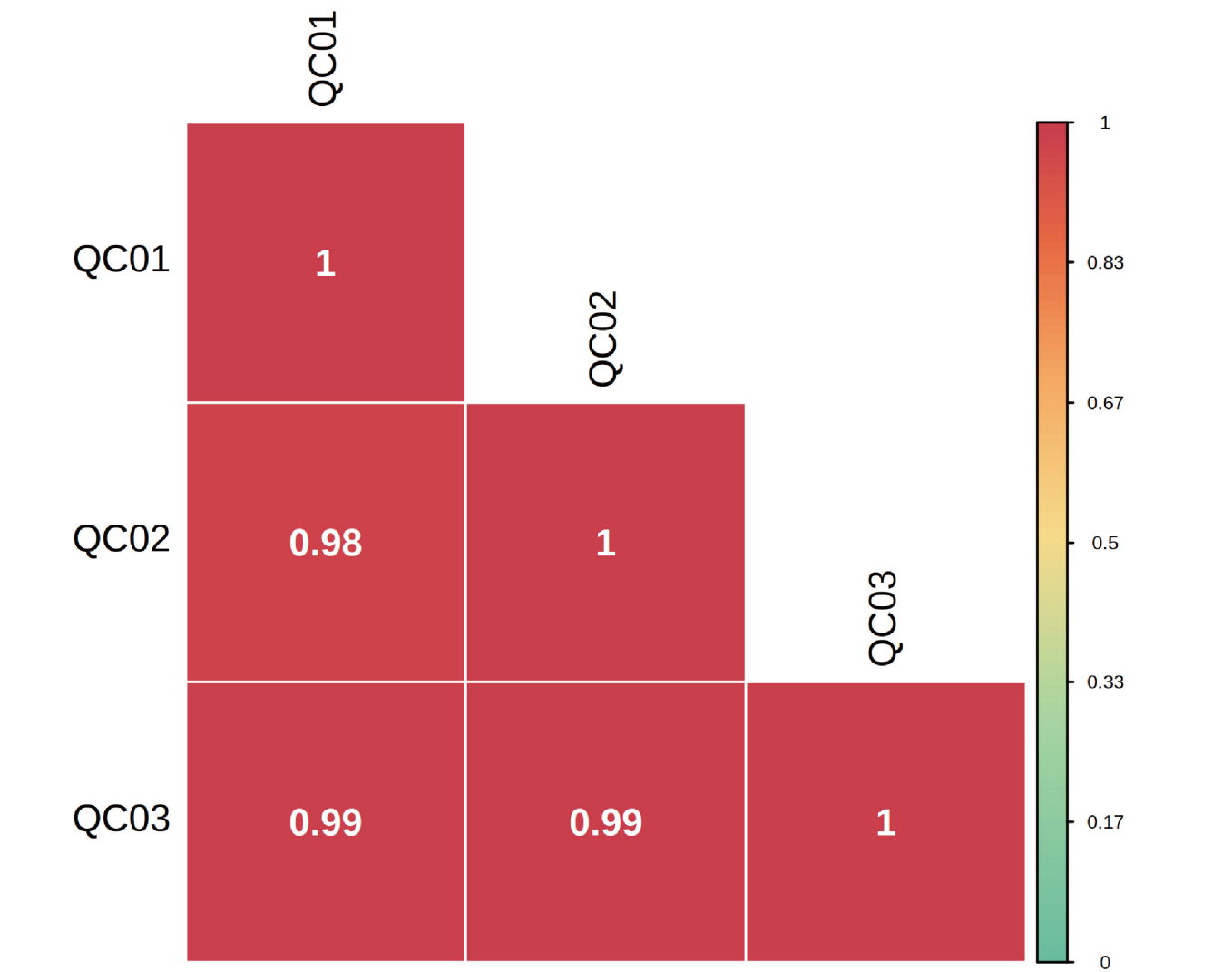

B

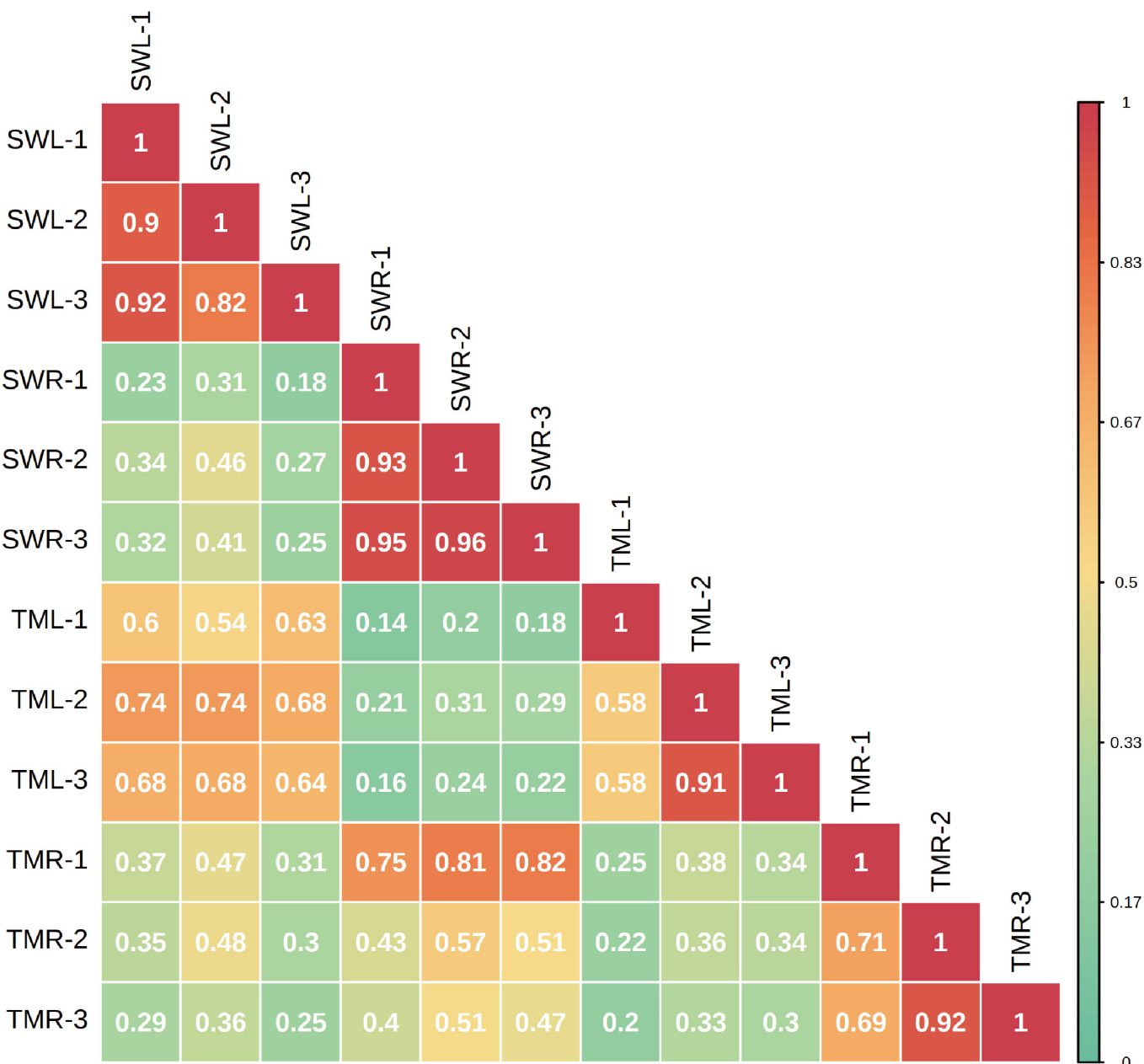

Supplement: Supplemental Information 7 [file peerj-14-20722-s007.pdf]
